# A Rare Occurrence of Phantom Tongue Pain

**DOI:** 10.7759/cureus.28841

**Published:** 2022-09-06

**Authors:** Grant M Drake, Eric J Lam, Benjamin W Cooper

**Affiliations:** 1 Physical Medicine and Rehabilitation, Larkin Community Hospital, South Miami, USA; 2 Physical Medicine and Rehabilitation, Good Samaritan Hospital, West Islip, USA; 3 Internal Medicine, Aventura Hospital and Medical Center, Aventura, USA

**Keywords:** neuropathic pain, neuroplasticity, amputation, glossectomy, phantom pain

## Abstract

This is a case of phantom tongue pain observed in a 65-year-old male with a history of adenoid cystic carcinoma with involvement of the base of the tongue and supraglottic laryngopharyngeal cancer who underwent a laryngopharyngectomy and glossectomy for treatment. The patient subsequently developed phantom tongue pain in acute rehabilitation. Post total glossectomy phantom pain is rare, and as this is a singular appendage, current techniques that rely on the presence of an intact limb, such as mirror therapy could not be applied to our patient. Therefore mental imagery techniques originally developed for extremity amputation required adaptation to the context of total glossectomy. Recommended anticonvulsant medications, desensitization, and mental imagery techniques for phantom limb pain were effective in relieving the patient’s phantom tongue pain. Utilizing therapeutic desensitization techniques may allow for the direction of neuroplasticity in order to decrease pain.

## Introduction

The incidence of phantom limb pain (PLP) varies in literature but may be as high as 95 percent [1-4]. Phantom pain is typically described as burning, aching, or electrical shooting pain in the amputated limb [1]. Its mechanism is poorly understood. There are several accepted pharmacological therapies that can be effective in relieving phantom pain.

Phantom tongue pain can be treated using a variety of pharmacological interventions; however, the clinically relevant outcomes in studies, like pain, mood, function, depression, and quality of life vary based on the treatment provided [5]. Morphine continues to be an effective drug of choice for short-term analgesia. Ketamine and gabapentin appear to be beneficial [5]. Mirror therapy is one novel type of treatment that has been used with success in treating phantom limb pain [6]. Although phantom pain might be eliminated with these therapies, the phantom sensation may persist despite the use of pharmacological interventions.

Phantom tongue pain is a condition that is rarely reported, with there being only one known case in literature, published back in 1979 [7]. Our case uses currently accepted practices for the treatment and management of phantom limb pain and applied them to our rare case of phantom tongue pain. This case was previously presented as a poster presentation at the Physiatry 2021 conference on February 11th, 2021.

## Case presentation

A 65-year-old Hispanic male was admitted to our acute inpatient rehabilitation facility for functional decline. The patient had been diagnosed with adenoid cystic carcinoma involving the base of the tongue and supra-glottic larynx several months prior. He underwent total laryngopharyngectomy, glossectomy, and neck dissection with reconstructive thigh flap placement approximately one week prior to admission to our facility. A percutaneous endoscopic gastrostomy (PEG) tube had been placed, and he was to remain nothing by mouth until his surgical follow-up.

On admission, the patient was aphonic but able to communicate by dry-erase board. His primary complaint was severe neck pain, he denied fever, chill or other associated symptoms. Initial physical examination of the neck showed circumferential swelling and erythema. No evidence of purulence or drainage was visualized, the surgical site appeared well healing with no dehiscence. He experienced pain during neck rotation but was able to demonstrate a full range of motion.

He was receiving duragesic patch of 50 mcg q72h (every 72 h), percocet 5/325 mg q8h PRN (as needed) for breakthrough pain, and gabapentin 300 mg q8h. He was seen by the pain management consultation service and his analgesic regimen was adjusted, duragesic patch was discontinued and he was started on oxycontin 10 mg q12 with percocet 5/325mg 1-2 tablets q4h for breakthrough pain.

Several days later, his generalized neck pain was well controlled, however, in the interview, the patient described a sensation that his tongue was still present in his oropharynx and began to complain of throbbing tongue pain, initially at the 7-8/10 level per Numeric Rating Scale (NRS-11) numeric pain scale. Physical examination revealed only expected surgical changes in the oropharynx, laboratory investigations were unremarkable.

The patient’s gabapentin dosage was increased to 400 mg q8h and he began participating in mental imagery exercises with physical therapy consisting of mentally visualizing moving the tongue through a range of motion consisting of elevation, depression, and lateral movements, he would then visualize isometric holds of the tongue in each of those positions. He was to perform this for 10 repetitions with therapy and later while in bed up to once per hour. In addition, the patient was encouraged to repeat these mental imagery movements anytime pain sensations were felt on the phantom tongue.

The patient performed these mental imagery exercises with physical therapy and independently while in bed over the course of one week of his inpatient stay. At the end of his stay, the patient reported a decrease in pain to the level of 5/10 per NRS-11 numeric pain scale. 

## Discussion

Due to the complex nature of pain generation in the phantom pain patient, a multi-disciplinary approach to treatment is now preferred, including pharmacologic intervention and therapeutic modalities. Current therapeutic techniques for phantom limb pain include mirror therapy, desensitization techniques, graded motor imagery, or movement imagery training. Mirror therapy has been used with success and has been considered an effective adjunct to medical therapy for patients undergoing an amputation of their limb [8]. Mental imagery training is thought to activate similar neural pathways to actual movements [3]. The pharmacologic approach relies on medication classes traditionally utilized in the treatment of neuropathic pain such as anticonvulsants or tricyclic antidepressants. 

Addressing the complexity of phantom pain is an essential component of the amputation rehabilitation process. The more common amputations such as transmetatarsal, transtibial, transfemoral, or amputations involving the upper extremities have proven treatment approaches; whereas, treating singular appendage amputations, such as the tongue is still being researched. Within this nascent field of study, the number of patients with this presentation is quite small, which makes evaluating treatment modalities limited. Since our patient was without a tongue, mirror therapy was unable to be used. Therefore, we utilized accepted interventions for phantom limb pain discussed in the literature and applied them to phantom tongue pain [3]. Our patient’s treatment plan included desensitization and mental imagery, and its use contributed to the reduction of pain seen in our patient’s phantom tongue pain.

Phantom limb pain is a poorly understood pain syndrome with proposed central as well as peripheral neuropathic mechanisms. Investigational studies such as motor reorganization after upper limb amputation in man. A study with focal magnetic stimulation by Cohen et al. demonstrated neuroplastic changes in patient’s status post limb amputation [9]. Nerves reaching the site of amputation undergo axonotmesis, which may then lead to Wallerian degeneration. Additionally, collateral sprouting from fibers near the amputation site may also lead to hyperalgesia and other sensory changes including “expansion of peripheral receptive fields” [10]. The concept of being able to influence the remodeling of the somatosensory cortex with visual inputs allows for the basis that afferent stimuli to the cortex can bring about neurological change. Mental imagery, if vivid enough and regularly repeated, could be just as effective as mirror therapy.

The treatments used were able to have a short-term reduction in our patient’s phantom tongue pain. However, we were unable to assess long-term pain relief as our patient was lost to follow-up after leaving our acute inpatient rehabilitation facility. Therefore, assessing long-term pain relief using these modalities should be further investigated to determine overall effectiveness in relieving pain. An area for further research would be virtual reality models for bilateral limb amputations or tongue amputations that could provide visualization of the missing appendage, which is not possible with mirror therapy. It would be important to distinguish if mental imagery is as efficacious, if not more so, compared to visual inputs and to determine if there are any shortcomings to mental imagery in the treatment of phantom pain.

## Conclusions

Current therapeutic approaches such as mirror therapy and movement imagery training are thought to direct the neuroplastic potential of the amputee toward the remodeling of afferent and efferent pathways in favor of somatosensory “acceptance” of the altered anatomy. These approaches are typically performed in the context of limb amputation. In our case, movement imagery training was adapted to a singular appendage amputation and resulted in a decrease in the patient’s reported pain score. Novel approaches to alleviating phantom pain, like virtual reality, should be reevaluated for effectiveness in relieving pain. Moreover, there has been no general consensus on the optimal length of mirror therapy treatment. 

Therapeutic modalities to treat phantom tongue pain are quite limited when compared to phantom limb pain. There are not any accepted best practices for the treatment of phantom tongue pain due to the scarcity of cases. Taking advantage of the plasticity of the central and peripheral nervous system allows for therapies such as mirror therapy and repetitive mental imagery to promote a rewiring of the pain pathways involved in phantom limb pain and phantom tongue pain.
